# QT interval prolongation after sertraline overdose: a case report

**DOI:** 10.1186/1471-227X-5-5

**Published:** 2005-07-19

**Authors:** Rudolf A de Boer, Tonnis H van Dijk, Nicole D Holman, Joost P van Melle

**Affiliations:** 1 Department of Internal Medicine, Intensive Care Unit, Martini Hospital, Groningen, The Netherlands; 2 Department of Cardiology, University Medical Center Groningen, Groningen, The Netherlands

## Abstract

**Background:**

Selective serotonin reuptake inhibitors (SSRIs) are the most common antidepressants used in first-world countries and are generally well tolerated. Specifically, less cardiovascular toxicity has been reported in comparison with tricyclic antidepressants. Here we report QT interval prolongation after an overdose of the SSRI sertraline.

**Case presentation:**

A previously healthy female patient presented with an attempted suicide with overdoses sertraline (2250 mg), diazepam (200 mg), and temazepam (400 mg). Routine laboratory studies were normal and her ECG upon admission showed a normal QT interval. The next day, her ECG showed prolongation of the QT_c _interval up to 525 ms. After discontinuation of sertraline the QT interval normalized. Echocardiography and exercise electrocardiography were normal. After hospitalization, the patient resumed sertraline in the normally recommended dose and QT interval remained within normal ranges.

**Conclusion:**

It seems that the SSRI sertraline in overdose may cause QT interval prolongation.

## Background

Since their introduction in 1987, the use of Selective Serotonin Reuptake Inhibitors (SSRIs) has increased dramatically [1]. They clearly have a more favorable safety profile compared to tricyclic antidepressants [2], although prolongation of the QT interval has been reported as a side effect [3]. This is an important side effect since prolongation of the QT interval is strongly associated with life-threatening arrhythmias, most notably torsades de pointes. Although sertraline belongs to the same class of antidepressants, controversy persists whether this holds true for the SSRI sertraline [4]. Here we here present a patient with prolonged QT interval after sertraline overdose.

## Case presentation

A 40-year old female patient was referred to our emergency department because of an intended overdose with 200 mg diazepam, 400 mg temazepam, and 2250 mg sertraline.

Her main complaints were fatigue and drowsiness. Blood pressure, pulse rate, and auscultation of the heart and lungs were normal. The patient was treated with sodiumsulfate and charcoal and was admitted to the intensive care unit for continuous control of vital signs. Routine laboratory studies (hematology, chemistry) were normal. Plasma levels of diazepam and temazepam were elevated, 1155 ugr/l (normal: 125 – 750 ugr/l) and 1710 ugr/l (normal: 300–900 ugr/l, respectively). Plasma levels of sertraline and desmethylsertraline were 174 ug/l (normal 20–55 ug/l [5]) and 276 ng/l, respectively.

Her ECG upon admission (upper panel of the figure) shows a sinus rhythm (77 b.p.m.) without conduction disturbances. QT interval in lead V2 was 370 ms. We used the Bazett method (QT time divided by the square root of the RR interval) to calculate the corrected QT (QT_c_). QT_c _at admission was 420 ms and negative T-waves were found in leads V1–V3. A second ECG, taken one day after admission (lower panel of the figure), showed a markedly prolonged QT interval with deepened negative T waves in leads V1–V3. QT interval was 520 ms in V2, at a heart rate (HR) of 63 b.p.m (QT_c _525 ms). An old ECG (august 2002) showed a sinus rhythm with a HR of 63 b.p.m and a QT interval in lead V2 of 370 ms (QT_c _373 ms; ECG not shown).

After 4 days the patient was discharged to a psychiatric hospital because the risk for another suicide attempt was deemed high by the psychiatric consultant. After discharge, the patient underwent further out-patient cardiac evaluation. Echocardiography revealed no structural heart disease. On exercise electrocardiography, patient reached 88% of her maximum HR – no abnormal ST-segment changes were observed. Hereafter, the use of sertraline was resumed in a dose of 50 mg twice daily under guidance of her psychiatrist. Control ECG revealed a normal QT interval (not shown).

## Discussion

We here present a patient with prolonged QT interval associated with sertraline overdose. An acquired cause of QT prolongation was suspected since QT intervals had been normal on admission, about 3 hours after ingestion of 2250 mg of sertraline (11 times the maximum maximum recommended dose of 200 mg/day), and were markedly prolonged after one day in hospital. The QT interval normalized after sertraline withdrawal. Therefore, a temporal relation existed between the overdose of sertraline and the development of QT prolongation. However, other causes for QT prolongation, both acquired and inherited, must be considered. For example, combinations of psychoactive drugs have been shown to cause prolongation of the QT interval [6], and our patient ingested temazepam as well as nitrazepam in overdose.

Whereas previous clinical studies [7-10] did not reveal any QT prolongation as a side-effect of sertraline, this case report suggests it may have this potential. We are aware of 1 additional report by Amin et al [11] who described 'a clinically significant' increase in QT interval after treatment with 200 mg of sertraline, however the magnitude of QT prolongation was not specified.

Naturally, implications of this finding are limited because it is only a single case. Two other limitations deserve comment. First, we did not perform a rechallenge with high dosage of sertraline, since this would be unethical. Second, only one blood sample was taken to assess plasma concentration of sertraline – the sertraline plasma level was found clearly increased according to other reports [5,12]. It was therefore not possible to investigate the relation between the course of QT interval prolongation and their paralleled serum levels of sertraline

## Conclusion

Our observation suggests that the SSRI sertraline may have the potential to prolong QT interval in rare cases. This case underscores the need for continuous post marketing surveillance.

## List of abbreviations

HR heart rate

LV left ventricular

QT_c _Corrected QT interval

SSRI selective serotonin reuptake inhibitor

## Competing interests

The author(s) declare that they have no competing interests.

## Authors' contributions

RADB, THVD, and NDH cared for the patient in the intensive care unit, conducted QT analyses, and arranged laboratory samples. RADB and JPVM noticed that QT interval prolongation had not been discussed previously in the case of sertraline overdose. RADB, THVD, NDH wrote the paper, whereas JPVM critically revised the discussion for important intellectual content. All authors read and approved the final manuscript.

**Figure 1:**
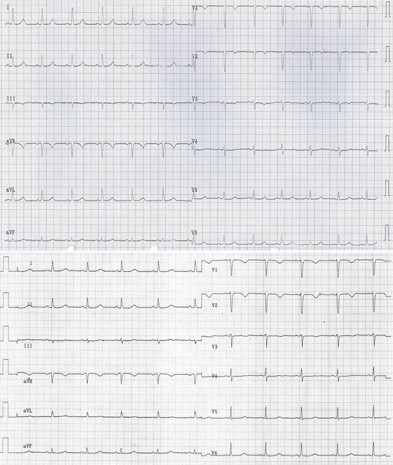
ECGs of the patient. ECG of the patient upon admission (upper panel) shows a normal sinus rhythm with a QT interval in lead V2 of 370 ms (QT_c _420 ms). There were negative T-waves in leads V1–V3. A second ECG was obtained one day after admission (lower panel) shows a markedly prolonged QT interval of 520 ms in V2 (QT_c _525 ms).

## Pre-publication history

The pre-publication history for this paper can be accessed here:


